# Preference for Orientations Commonly Viewed for One’s Own Hand in the Anterior Intraparietal Cortex

**DOI:** 10.1371/journal.pone.0053812

**Published:** 2013-01-07

**Authors:** Regine Zopf, Mark A. Williams

**Affiliations:** Department of Cognitive Science, Macquarie University, Sydney, New South Wales, Australia; University of Reading, United Kingdom

## Abstract

Brain regions in the intraparietal and the premotor cortices selectively process visual and multisensory events near the hands (peri-hand space). Visual information from the hand itself modulates this processing potentially because it is used to estimate the location of one’s own body and the surrounding space. In humans specific occipitotemporal areas process visual information of specific body parts such as hands. Here we used an fMRI block-design to investigate if anterior intraparietal and ventral premotor ‘peri-hand areas’ exhibit selective responses to viewing images of hands and viewing specific hand orientations. Furthermore, we investigated if the occipitotemporal ‘hand area’ is sensitive to viewed hand orientation. Our findings demonstrate increased BOLD responses in the left anterior intraparietal area when participants viewed hands and feet as compared to faces and objects. Anterior intraparietal and also occipitotemporal areas in the left hemisphere exhibited response preferences for viewing right hands with orientations commonly viewed for one’s own hand as compared to uncommon own hand orientations. Our results indicate that both anterior intraparietal and occipitotemporal areas encode visual limb-specific shape and orientation information.

## Introduction

Behavioural experiments have consistently provided evidence that viewing the body modulates a multitude of important processes for human beings. Visual information regarding bodies supports social perception, such as recognizing other individuals [1] including their emotions [2], actions and goals [3]. In addition, viewing the body also modulates sensory processing related to one’s own body and the surrounding space for example of touch [4], [5], pain [6], [7] and nearby visual stimuli [8], [9].

Neuroimaging studies have identified occipitotemporal, fusiform as well as parietooccipital (posterior intraparietal sulcus, V3A, V7) areas in the human brain which specialize in visual processing of human bodies [10], [11] (see [12], [13] for reviews). Activity in these areas is significantly greater when participants view images of bodies or body-parts as compared to a wide range of other visual stimulus categories such as faces and objects. Furthermore, body-part specific representations, for example of the human hand, have been shown to be dissociable from other body-parts [14], [15].

Another line of research into neural body-related information processing suggests that other cortical areas, such as the anterior intraparietal and premotor areas, specialize in encoding of visual events in space near the hand (peri-hand space) [16], [17]. Neurons in intraparietal and premotor areas encode and integrate information regarding visual, tactile and auditory stimuli which are nearing and/or touching the body [17], [18], [19], [20]. These areas have also been related to the experience of body ownership which can be induced for artificial hands in the famous ‘rubber hand illusion’ [20]. Motivated by these findings, hand ownership in the rubber hand illusion has also been linked to peri-hand space mechanisms [21], [22], [23].

Research regarding sensory processing in near-body space in humans has been motivated by comparable response characteristics of individual neurons in premotor and parietal regions of nonhuman primates [24], [25], [26], [27], [28], [29], [30]. Interestingly, both in humans and monkeys, neural activity in premotor and parietal areas was also modulated by visually presented hands even if these were not their own [17], [20], [27], [29].

In humans, both viewing a hand [16], [17] as well as the orientation of the viewed hand [20] modulates neural responses to visual or multisensory events in intraparietal and premotor areas. For example, in one study participants viewed a red ball which appeared either near or far from the participant’s visible hand. Significant adaptation effects for viewing the ball near the hand were found in the middle and inferior occipital sulcus, the anterior intraparietal sulcus, the bilateral supramarginal gyrus, and the dorsal and ventral premotor areas [16]. Further, Ehrsson et al. (2004) had participants view a rubber hand which was placed in two different orientations (upwards [i.e. fingers pointing up/congruent to the real hand] or downwards [i.e. fingers pointing down towards the lying subjects/incongruent to real hand]) and either touched synchronously or asynchronously with the hidden real hand (i.e., methods typically used in the rubber hand illusion) [20]. The authors found that an anterior intraparietal area encoded visual information regarding the viewed hand orientation (upward preference) as well as information regarding the synchrony of touch and vision (preference for synchronous multisensory stimuli). Interestingly, viewing the hand in an upward/congruent orientation in combination with synchronous multisensory information preferentially activated the premotor cortex. These studies suggest that visual information regarding the viewed hand might be represented and integrated with other sensory information in anterior intraparietal and ventral premotor cortices.

In fMRI studies investigating near-body space, participants typically viewed a hand together with other visual or multisensory stimuli. These stimuli were either near and/or touching the hand. In the present study we tested whether in addition to occipitotemporal hand-specific areas, also anterior intraparietal and ventral premotor activation in peri-hand areas is increased for visual information of hands as compared to other stimulus categories. We found processing preferences for static hands and feet images in the left anterior intraparietal area. Furthermore, we investigated response sensitivity to viewed hand orientation in occipitotemporal, intraparietal and premotor hand-related areas. We predicted that peri-hand regions would be especially responsive to viewing orientations commonly viewed for one’s own hand. We found significantly increased responses to common hand orientations viewed for one’s own right hand in anterior intraparietal and also in occipitotemporal areas in the left hemisphere.

## Methods

### Participants

Twenty-one right-handed (self-reported) participants took part in the first experiment. We excluded the data of one participant from analysis due to excessive head movement (>3 mm across the scan). The remaining group consisted of 13 females and 7 males (mean age = 26.0 years, SD = 6.5 years).

Seventeen new right-handers (self-reported) participated in the second experiment. We excluded the data of two participants because of excessive head movement (>3 mm across the scan). This resulted in a group consisting of 15 participants (8 female, mean age = 26.5 years, SD = 8.4 years).

### Ethics Statement

All participants gave their written informed consent to participate prior to the start of experiments. The experiments were conducted in accordance with ethical standards laid down in the 1964 Declaration of Helsinki and were approved by the Macquarie University Ethics Review Committee. Participants received $20 per hour for their participation.

### MRI Data Acquisition

The MRI data were acquired using a Siemens Verio 3T scanner in combination with a 32-channel head coil (Erlangen, Germany). Gradient echo T2*-weighted echo planar imaging (EPI) was implemented for functional imaging. One scan volume was obtained every 3 seconds (TR) and consisted of 32 slices (TE = 37 ms, FOV = 200×200 mm, in-plane resolution 1.79×1.79 mm, slice thickness 2.5 mm, interslice gap 0.5 mm, flip angle = 90°). The axial slices were aligned with the aim to cover occipitotemporal, parietal and frontal brain areas. The first four volumes in each run were automatically discarded. In Experiment 1 each run comprised 171 recorded volumes and in Experiment 2 each run contained 104 volumes.

For each participant also a high-resolution structural image was acquired (3D-MPRAGE sequence, voxel size 0.94 isotropic, FOV: 240×240 mm, 176 slices, TR = 2110 ms, TE = 3.54 ms, flip angle = 9°).

### Experimental Setup

During brain scanning, participants lay comfortably in a supine position on the MRI table. One of their hands was positioned on a Lumina response box placed on the side of the body. In Experiment 1 this was always the right hand; in Experiment 2 the hand used for responses (left or right) was counterbalanced across participants. The presentation of stimuli was controlled using Presentation software (Neurobehavioral Systems, Albany, CA, USA; http://www.neurobs.com/). The images were presented using a 15-inch Macintosh Power Book and projected onto a screen positioned ∼1.2 m behind the participant’s head. The participants viewed the screen via a mirror mounted ∼15 cm above the eyes.

### Stimuli and Experimental Design- Experiment 1

**Figure 1:**
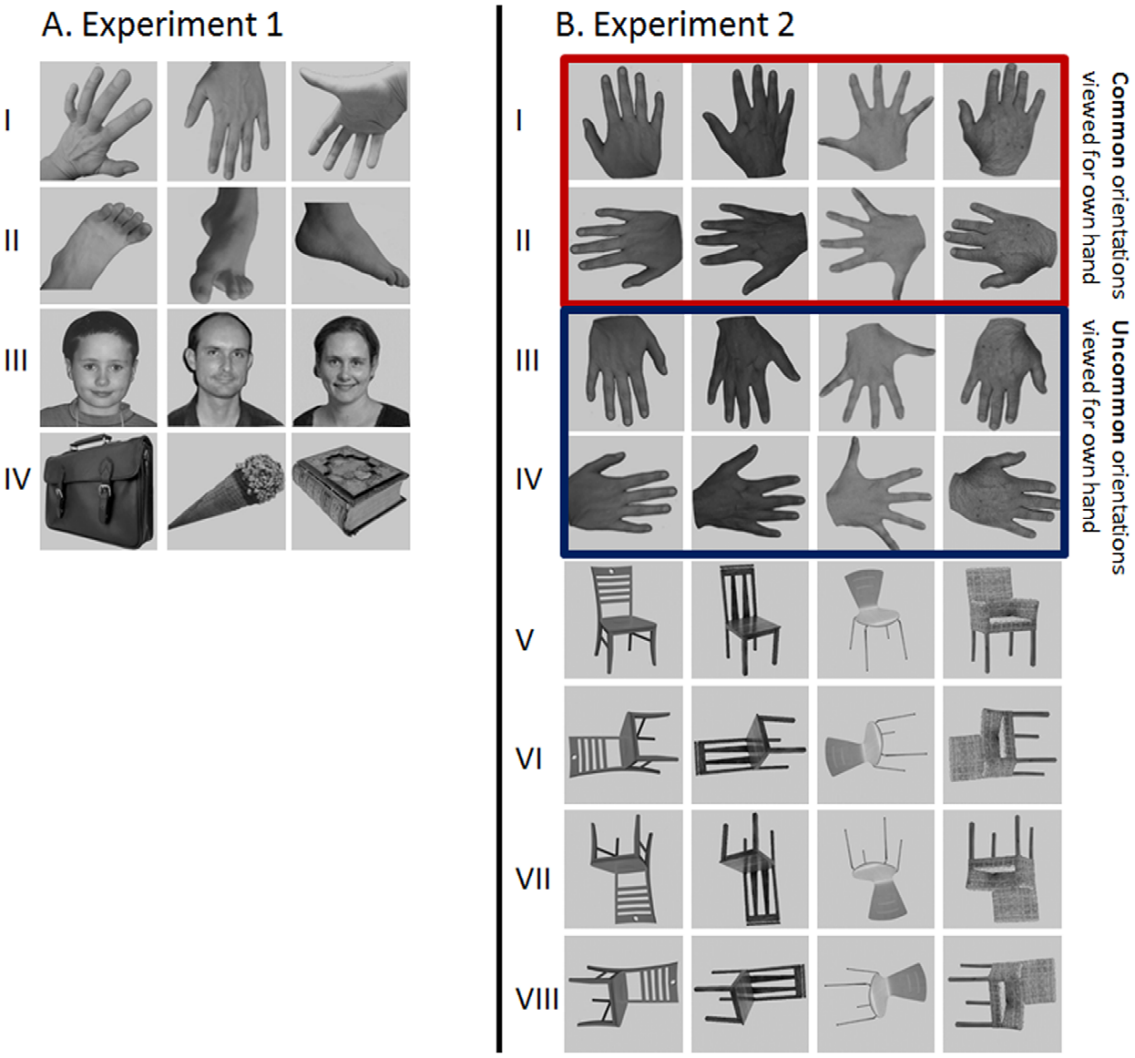
Examples of stimuli presented to participants. Please note that the face images and some hand images are only similar to the images we used and not original images. A) Experiment 1. Each category comprised 96 stimuli. Half the hand (I) and feet (II) stimuli pictured a right body part and the other half a left body part. A mix of different viewpoints and orientations were shown. Half of the face stimuli (III) depicted female and the other half male individuals. A wide range of different objects (IV) was presented. B) Experiment 2. Each condition comprised 128 stimuli. The design included two conditions with orientations commonly viewed for one’s own right hand (upwards [I], leftwards [II]) and two orientations which are not typical or uncommon for one’s own right hand (downwards [III], rightwards [IV]). Furthermore, four conditions were incorporated with views of chairs in four different orientations (upwards [V], leftwards [VI], downwards [VII], rightwards [VIII]).

In the first experiment, participants viewed images belonging to one of four categories: hands, feet, faces and objects (Figure 1A). All hands, feet and face images depicted other persons who did not take part in the study. Please note that the face images and some hand images used in the figures are not the original images used in the study, but instead are similar images used for illustrative purposes only. The subjects in the photographs have given written informed consent, as outlined in the PLOS consent form, to publish their photographs. Each stimulus category comprised 96 grey-scale images. The stimuli subtended a visual angle of about 7 degrees and were presented centrally.

An fMRI block-design was implemented. Visual stimuli for one category were shown within epochs of 16 seconds (16 images per epoch) and each image was presented for 500 ms (500 ms inter-stimulus interval). The data were obtained in three runs; each run lasted 8 minutes and 16 seconds (31 epochs) and started with a fixation epoch, followed by one epoch for each stimulus category and again one fixation epoch. This was repeated six times in each run, which resulted in 18 epochs per category condition across all three runs. The 96 stimuli were presented in random order and only shown once in each run. The category condition order was pseudo-randomized across participants so that the likelihood for each condition to be at a certain position was similar. Furthermore, for the last nine participants, we also varied the condition order across runs (second run backward order and third run split half order; i.e. last two conditions in the first run were presented first in the third).

Participants were instructed to fixate a red cross presented in the centre of the screen and to press a button when the presented image flickered. One of the sixteen images in each epoch was turned off very briefly (for 50 ms). Participants correctly identified this flicker in 84% (SD = 15.2) of cases across all conditions with an average response time of 778 ms (SD = 76.8). Repeated measures analyses of variance (ANOVA) confirmed that there was no significant performance difference across conditions.

### Stimuli and Experimental Design- Experiment 2

In the second experiment we aimed to investigate the effect of viewed hand orientation on neural responses. Participants viewed images of hands and chairs in four different orientation conditions (Figure 1B). We included two orientations which are typical or common for one’s own right hand (i.e., viewing the back of the right hand with the finger pointing upwards or leftwards) and two orientations which are uncommon for one’s own right hand (i.e., viewing the back of the right hand with the finger pointing downwards or rightwards). These latter two orientations are difficult to produce with one’s own right hand due to anatomical constraints. Note that the same stimuli were used for each of the four orientation conditions but rotated by multiples of 90 degrees. In this experiment, we used chairs for the object category in order to approximately match the level and number of exemplars. Chairs have previously been used in contrast to hands and have a comparable structure [14]. Each of the eight conditions comprised 128 grey-scale images. The stimulus location varied around the fixation cross in order to avoid possible stimulus orientation and position confounds.

Again an fMRI block-design was implemented. The data were obtained in eight runs each comprising 19 epochs. Each run lasted 5 minutes and 4 seconds and started with a fixation epoch, followed by one epoch for each stimulus category and again one fixation epoch. The category and the orientation of the stimuli were constant within one epoch. Each condition was repeated twice within each run, which resulted in 16 epochs per condition across all eight runs (three participants completed only six experimental runs because of technical problems). The 128 stimuli per condition were presented in random order and repeated two times across all runs. The condition order was randomized for each run.

The participants were instructed to fixate a red cross presented in the centre of the screen and to press a button (eight participants with the right hand and seven participants with the left hand) when the image flickered. One target was presented per epoch in a random position. Participants correctly identified the target in 79% (SD = 15.5) of cases across all conditions with an average response time of 804 ms (SD = 92.3). Repeated measures ANOVA confirmed that there was no significant performance difference across conditions.

### Data Analysis

The data were analysed with SPM8 (Wellcome Department of Cognitive Neurology, London, U.K., http://fil.ion.ucl.ac.uk/spm/). First, all obtained volumes per participant were realigned to correct for small head-movements. Better spatial normalisation results were obtained with the structural image. Therefore, a mean of the functional volumes was co-registered to the participant’s structural image. The structural image was segmented and normalized to an MNI (Montreal Neurological Institute) template included in SPM. The obtained transformation parameters were then applied to the co-registered functional volumes, which were re-sampled to a 2×2×2 mm voxel size. The spatially transformed functional data were spatially smoothed using a 6 mm FWHM isotropic Gaussian kernel. A general linear model (GLM) was fitted to the data with four regressors in Experiment 1 (hands, feet, faces, objects) and 8 regressors in Experiment 2 (hand-upwards, hand-leftwards, hand-downwards, hand-rightwards, chair-upwards, chair-leftwards, chair-downwards, chair-rightwards) for each run separately. Each condition was modelled with a boxcar function and convolved with the SPM’s canonical hemodynamic response function. Furthermore, to remove low-frequency drifts in Experiment 1 the default temporal high-pass data filter cut-off (128 seconds) was employed, whereas in Experiment 2, due to the increased number of conditions, a customised high-pass filter cut-off of 288 seconds (twice condition cycle) was used.

We defined intraparietal and premotor regions of interest (ROIs) in both hemispheres based on means across published coordinates in peripersonal space and body-ownership studies [16], [17], [20]. The mean MNI coordinates were (−33,−50,55) for the left intraparietal area, (35,−43,50) for the right intraparietal area, (−54,8,26) for the left ventral premotor area and (47,11,29) for the right ventral premotor area (see Table S1 for more details). We then defined a sphere with 5 mm radius around the mean coordinates using WFU Pickatlas, version 2.5 ([31]; http://www.fmri.wfubmc.edu/cms/software/). We also created ROIs around the mean coordinates of previously reported hand-selective occipitotemporal regional peaks in the left (−46,−69,−1) and right hemisphere (48,−64,−1). In previous studies these occipitotemporal areas exhibited response preferences for body visual stimuli especially for hand/upper limb and in the left hemisphere ([14] (Ref. Table 1 contrast Hands>Bodyparts), [15] (Ref. Table 1)).

For each ROI we then obtained average condition-specific estimated signal changes using GLM parameter estimates (betas). For statistical analysis of Experiment 1, we conducted a repeated-measures ANOVA with three factors (3×2×4): ROI with three levels (intraparietal, ventral premotor and occipitotemporal), hemisphere with two levels (left and right) and stimulus category with four levels (hands, feet, faces and objects). In addition, we planned to investigate the effect of viewed stimulus category for each ROI separately and conducted repeated-measures ANOVA for each ROI (2×4) including the factors hemisphere and stimulus category.

For Experiment 2 the repeated-measures ANOVA comprised four factors (3×2×2×2): ROI (intraparietal, ventral premotor and occipitotemporal), hemisphere (left hemisphere and right hemisphere), stimulus category (hands and chairs) and orientation (upwards/leftwards and downwards/rightwards). The orientations commonly viewed for one’s own hand comprised the within-subject averages for upwards and leftwards orientations; whereas orientations uncommonly viewed for one’s own hand comprised downwards and rightwards orientations. For the chair category we used the matching orientations. Again, we analysed differences in estimated signal changes for each ROI separately with repeated-measures ANOVA (2×2×2) including the factors hemisphere, stimulus category and orientation.

When reporting our results we focus on effects involving the factor stimulus category because only these effects are of theoretical interest for our present study.

## Results

### Experiment 1

**Figure 2:**
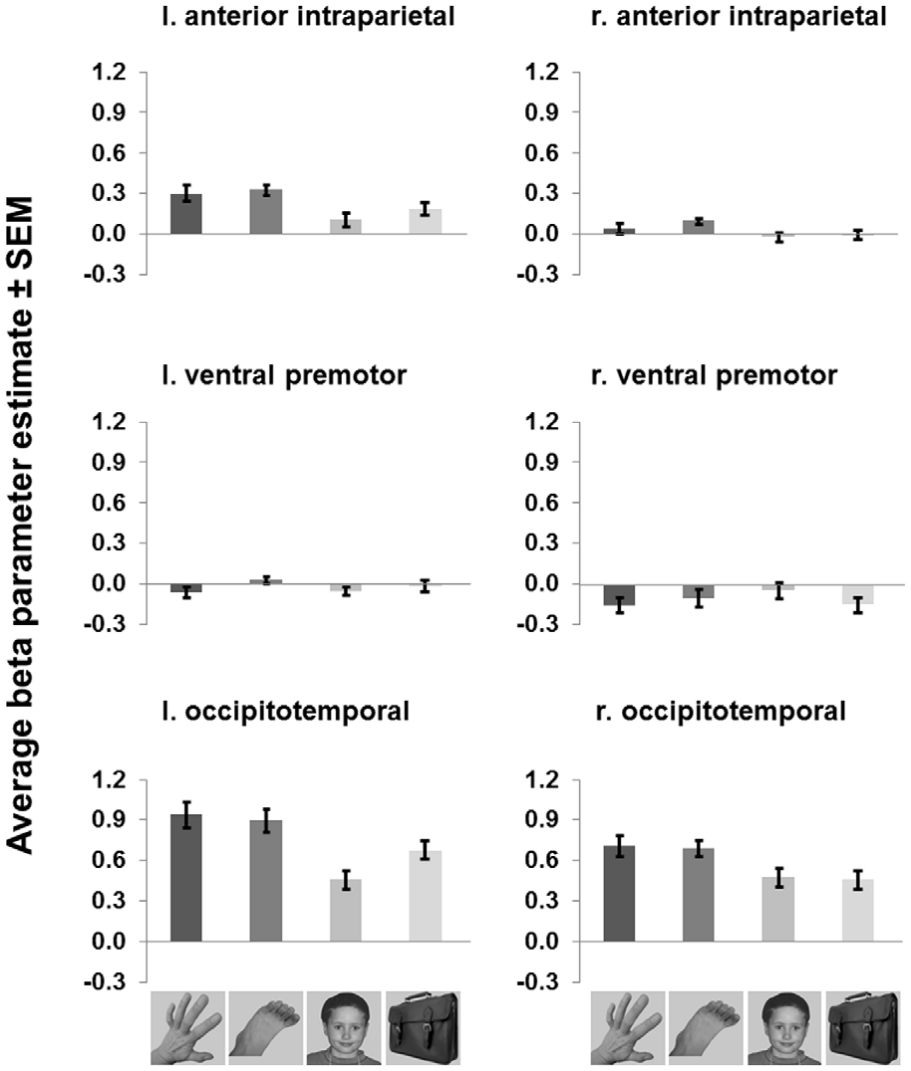
Experiment 1: Average parameter estimates (betas) for anterior intraparietal, ventral premotor and occipitotemporal ROIs in left (l.) and right (r.) hemisphere. Error bars represent within-subjects SEM.

The three-way ANOVA comprising the factors ROI, stimulus category and hemisphere resulted in a significant main effect of stimulus category (*F*[3,57] = 2.99, *p* = .039). Stimulus category interacted significantly with the factor ROI (*F*[6,114) = 4.42, *p*<.001) which reveals differences in sensitivity to viewed stimulus category between the three ROIs. Furthermore, stimulus category interacted significantly with the factor hemisphere (*F*(3,57) = 5.29, *p* = .003). The differences between viewed body-extremity and other categories are most pronounced for the intraparietal and occipitotemporal ROIs and especially in the left hemisphere (Figure 2). None of the other interactions with stimulus category were significant.

Next we conducted a two-way ANOVA comprising the factors hemisphere and stimulus category for each ROI separately. This revealed a significant main effect of stimulus category for the anterior intraparietal ROI (*F*[3,57] = 3.66, *p* = .018). In line with our hypothesis, this suggests that the anterior intraparietal area is sensitive to visual category information contained in static images. Furthermore, estimated signal changes for viewing hands and feet are significantly larger as compared to viewing objects and faces (post-hoc comparison, *t*(19) = 2.66, *p* = .031, one-sided, Bonferroni-corrected p-value). This was especially the case for the left hemisphere (Figure 2), however the interaction between hemisphere and stimulus category was not significant for this ROI (*F*[3,57] = 1.75, *p* = .167). We performed three further one-sided post-hoc comparisons to test if the average estimated signal change in the intraparietal region (across both hemispheres) for the hands condition is larger as compared to the other three conditions. However, none of these individual comparisons was significant after Bonferroni-corrections (all *p*>.05).

In contrast to the anterior intraparietal ROI, the ANOVA results did not show any significant effect for the ventral premotor area (all *p*>.05). Consistent with previous studies [14], [15] we found a significant main effect of stimulus category for the occipitotemporal ROI (*F*[3,57] = 4.67, *p* = .005). For this ROI, stimulus category interacted significantly with hemisphere (*F*[3,57] = 5.18, *p* = .003). Post-hoc comparisons revealed that for both hemispheres viewing hands and feet resulted in increased BOLD signal changes as compared to faces and objects (left hemisphere: *t*(19) = 3.97, *p* = .001, right hemisphere: *t*(19) = 3.16, *p* = .008, one-sided, Bonferroni-corrected p-values). A further post-hoc test revealed that this response increase for viewing hands and feet was more pronounced in the left hemisphere as compared to the right hemisphere (*t*(19) = 2.33, *p* = .046, one-sided, Bonferroni-corrected p-value).

To summarize Experiment 1, we found ROI-dependent effects of viewed stimulus category. For the anterior intraparietal and occipitotemporal, but not the ventral premotor ROI, we found evidence for neural sensitivity to viewed object category and increased responses to viewing hands and feet. Furthermore, our results indicate stronger stimulus category sensitivity for the ROIs in the left hemisphere.

### Experiment 2

The aim of Experiment 2 was to investigate the interaction between stimulus category and orientation. Participants viewed images of two different categories (hands and chairs) in different orientations (Figure 1B).

**Figure 3:**
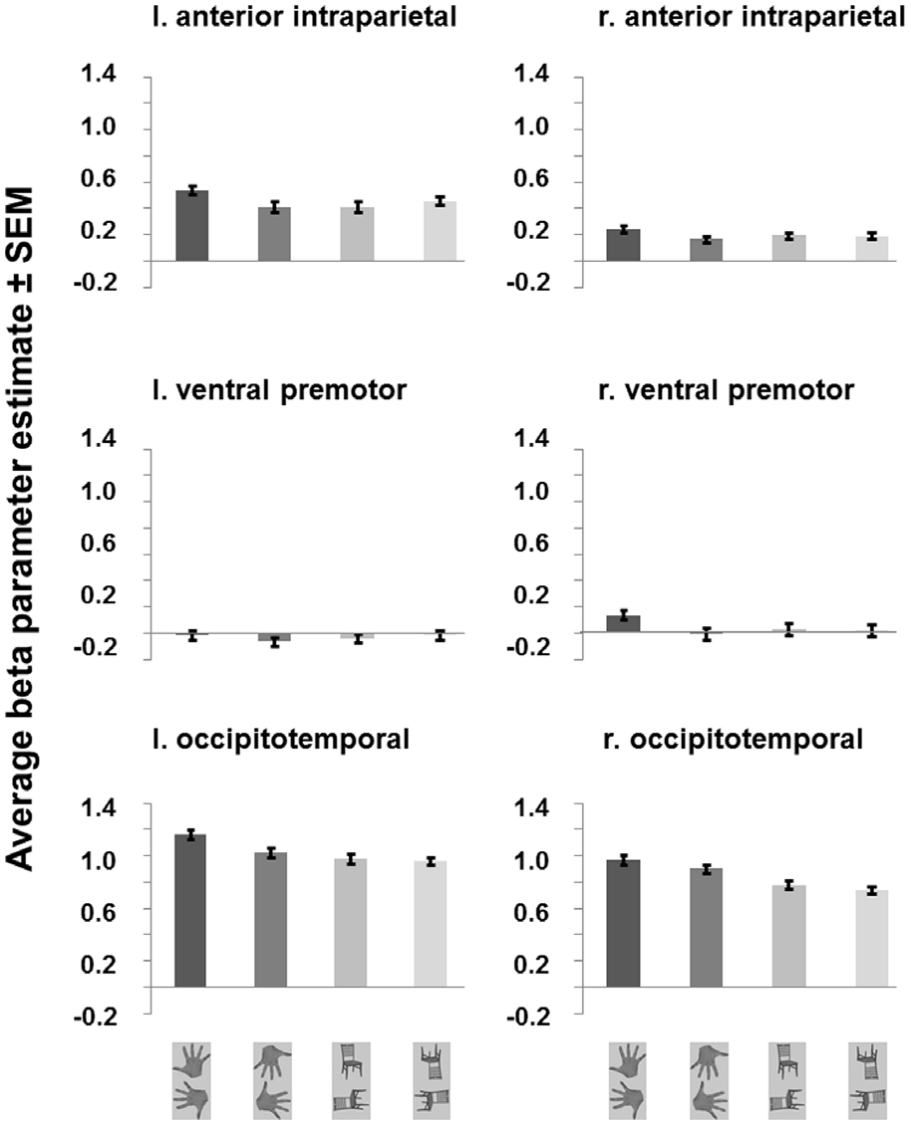
Experiment 2: Average parameter estimates (betas) for anterior intraparietal, ventral premotor and occipitotemporal ROIs in left (l.) and right (r.) hemisphere. Error bars represent within-subjects SEM.

First, we report the results of the four-way ANOVA comprising the factors ROI, hemisphere, stimulus category and orientation. We did not obtain a significant main effect of stimulus category (*F* [1], [14] = 3.27, *p* = .092). In line with Experiment 1, a significant interaction of stimulus category with ROI (*F* [2], [28] = 8.67, *p* = .001) revealed differences between the ROIs in response to different stimulus categories (hands versus chairs) across all orientations. We found a significant interaction between stimulus category and orientation (*F* [1], [14] = 4.94, *p* = .043). This interaction reveals that the effect of orientation was strongest for the hand category. Viewing hands with orientations commonly viewed for one’s own right hand (upwards/leftwards) resulted in increased responses compared to viewing uncommon views (downwards/rightwards) and compared to viewing chairs in different orientations (Figure 3). None of the other interactions with stimulus category were significant.

Next we conducted a three-way ANOVA for each ROI separately comprising the factors hemisphere, stimulus category and orientation. In contrast to Experiment 1, for the intraparietal ROI, we did not find a main effect of stimulus category (*F* [1], [14] = .572, *p* = .462). However, we found a trend for the stimulus category and orientation interaction (*F* [1], [14] = 4.00, *p* = .065) and a significant three-way interaction between hemisphere, stimulus category and orientation (*F* [1], [14] = 5.40, *p* = .036). Viewing hands common to one’s own right hand (upwards/leftwards) resulted in larger signal changes as compared to uncommon orientations (downwards/rightwards) as well as compared to chair conditions especially in the left hemisphere (Figure 3). Post-hoc comparisons for the left hemisphere revealed that viewing hands in common orientations resulted in significant larger responses contrasted to uncommon hand orientations (*t* [14] = 2.73, *p* = .016, one-sided, Bonferroni-corrected p-value) as well as compared to the chair category (all orientations; *t* [14] = 2.17, *p* = .048, one-sided, Bonferroni-corrected p-value).

As in Experiment 1, for the ventral premotor region we did not find a significant main effect of stimulus category nor a significant interaction with stimulus category (all *p*>.05). For the occipitotemporal ROI we found a significant main effect of category (*F* [1], [14] = 10.58, *p* = .006), a trend for the stimulus category and orientation interaction (*F* [1], [14] = 4.13, *p* = .062) and a significant three-way interaction between hemisphere, stimulus category and orientation (*F* [1], [14] = 5.58, *p* = .033). Similar to the anterior intraparietal ROI, viewing hands common to one’s own right hand (upwards/leftwards) resulted in larger signal changes as compared to uncommon orientations (downwards/rightwards) as well as compared to chair conditions especially in the left hemisphere (Figure 3). Post-hoc comparisons for the left hemisphere revealed that viewing hands in common orientations (upwards/leftwards) resulted in significant larger responses as compared to uncommon (downwards/rightwards) hand orientations (*t* [14] = 2.99, *p* = .003, one-sided, Bonferroni-corrected p-value) as well as compared to the chair category (all orientations; *t* [14] = 3.35, *p* = .010, one-sided, Bonferroni-corrected p-value).

It might be possible that the differences we found between commonly and uncommonly viewed hand orientations for one’s own hand are due to general differences in how often or commonly these are viewed. In this study only one chair orientation (upwards) is commonly viewed. Therefore, we have contrasted the single common chair view (upwards) with the average across the three uncommon chair views (leftwards, downwards, rightwards) employing six paired t-tests for both hemispheres and all three ROIs separately. None of these comparisons was significant (all *p*>.05, uncorrected for multiple comparisons). This indicates that the difference we found between common and uncommon own hand views is specific for hand orientations and not due to the possible general commonness of the views.

Furthermore, participants made responses involving the hand. Execution of hand movements has been shown to activate occipitotemporal and anterior intraparietal areas [15], [32]. Thus, it may be that responding with the hand interacts with the viewed hand orientation. A response was only required for one target per epoch (6.25% of all viewed stimuli) and the response requirements and performance did not differ between conditions. Furthermore, the hand-position relative to the body of the responding hand and the viewed hand differed (placed near the body versus viewed above the head). However, it may be possible, that stronger responses for the common hand view compared to the uncommon hand view reflect the alignment of participants’ own action with the visually presented hands. One subsequent prediction is that the orientation effects would be especially strong for participants who responded with their right hand (N = 8) as compared to participants who responded with the left (N = 7). Thus, we conducted additional ANOVA including the between-subject factor response hand. We did not find a significant interaction of response hand with any effects of interest. We also did not find a significant main effect for the between-subject factor response hand (all *p*>.05). This analysis does not support the notion of interactions between hand responses and viewed hand stimuli. However, we cannot completely rule out any potential interactions especially due to the small group sizes. An interesting question for future research is how proprioceptive information and action interact with viewed hand orientation in these areas.

To summarize, we found that stimulus category (hands versus chairs) interacted significantly with viewed orientation. This effect further interacted with hemisphere when analysing signal changes for individual ROIs. For anterior intraparietal and occipitotemporal ROIs in the left hemisphere we found significant differences in estimated fMRI signal changes for viewing hand orientations common for one’s own right hand as compared to uncommon views.

## Discussion

For the anterior intraparietal ‘peri-hand’ area our results indicate sensitivity to viewed stimulus category and response preferences for hand and feet compared to faces and object. In line with previous studies, hand and feet also elicited stronger responses as compared to faces and objects in the occipitotemporal hand-selective area [14], [15]. Furthermore, anterior intraparietal and occipitotemporal areas in the left hemisphere exhibited response preferences for viewing right hands with orientations commonly viewed for one’s own hand as compared to uncommon orientations. In contrast, we did not find any significant effects for the ventral premotor area. Across both experiments the sensitivity for category and hand orientation was more pronounced in the left as compared to the right hemisphere.

The current result is supported by previous electrophysiological work in humans suggesting the left intraparietal cortex exhibits hand-sensitive responses to static images [33]. In this study, neural activity was measured using intracranial surface electrodes in epilepsy-patients. The patients viewed images of several different categories including hands, faces and objects. Importantly, the authors report hand-selective responses which peaked at around 230 ms after stimulus-onset for intraparietal, occipitotemporal and frontal (possibly premotor) electrode sites.

Occipitotemporal and intraparietal areas also exhibit shape sensitivity for viewed non-body objects [34], [35], [36], [37], [38], [39]. For example Denys et al. (2004) employed fMRI in both humans and monkeys [37]. Increased activity in anterior intraparietal areas (among other areas) was observed for intact as compared to scrambled objects. Together with related findings using monkey neurophysiology and fMRI, this suggests that intraparietal areas can encode visual object shape. Our study give further support to the notion that a part of the anterior intraparietal cortex is especially sensitive to viewing hand-like shapes.

Importantly, results from our second experiment indicate that anterior intraparietal and occipitotemporal areas in the left hemisphere preferentially process orientations commonly viewed for one’s own right hand (Figure 3). Thus the anterior intraparietal and occipitotemporal areas in the contra-lateral hemisphere might represent visual information especially related to one’s own hand.

Previous research has shown that intraparietal and also occipitotemporal regions are involved in processing visual and multisensory events especially when near the hand [16], [17], [20]. Interestingly, anterior intraparietal and occipitotemporal areas have also exhibited response selectivity for observed and executed hand and foot movement ([32] includes further detailed overview of previous studies, [40], [41], [42], [43], [44]). And neural representations for observed and executed movement seem to differ in anterior intraparietal areas [32], [45] whereas cross-modal adaptation effects for observed and executed actions suggest common neural processing in the right inferior parietal lobule [46]. It is possible that representations of visual hand information in the intraparietal cortex support distributed and shared representations of observed and executed movements. Perhaps it is therefore not surprising that viewing richer and more dynamic stimuli of hand and foot movement as compared to static images increases activations in intraparietal and occipitotemporal areas [43]. Importantly, the current study demonstrates that both anterior intraparietal as well as occipitotemporal areas respond preferentially to visual information regarding extremeties presented in static images and in particular to hand orientations commonly viewed for one’s own contralateral hand.

A recent study showed that static images of hands and tools activated nearby and partly overlapping areas in the left occipitotemporal cortex [47]. Interestingly, in this study functional connectivity analyses suggest that activation in the left anterior intraparietal and the left premotor area is related to the activity in the occipitotemporal hand and tool region. Furthermore, in line with the current results from Experiment 1, viewing images of hands and tools as compared to animals and scenes led to significant increases in fMRI signal in the left anterior intraparietal area.

We suggest that regions in the occipitotemporal and anterior intraparietal cortex are dedicated to encoding sensory information, including visual information regarding one’s own hand, which is likely used to process stimuli closely related to one’s own body as well as to guide one’s own hand actions. One possibility is that visual information regarding the body and its orientation is useful to specify the location of one’s own body and the surrounding space (also see [27], [29] for relevant neurophysiological work in monkeys).

In contrast to our hypothesis, we did not find evidence for representations of static visual information of extremities in the premotor cortex. Visual information regarding the hand in combination with other multisensory information might be necessary to activate premotor cortex [17], [20]. Furthermore, premotor areas respond selectively to observed and executed hand movement [32], [40], [41], [43]. Thus, it could be that premotor areas are activated especially for more dynamic visual hand stimuli.

Our results suggest strong overlap between neural representations for hands and feet. We did not find significant differences for viewing hands versus viewing feet in intraparietal nor occipitotemporal areas, which might be due to shape or functional similarities between these two extremities. In contrast, previous studies showed significantly different responses to hands and feet (or upper limbs and lower limbs) in occipitotemporal areas exhibiting response preferences for the hand/upper limb [14], [15]. However, these studies also reported considerable overlap and common activation. For example Orlov at al. (2010) showed that for occipitotemporal areas with upper limb preference in fact lower limbs were the second-best preferred category compared to other body-parts. In addition hand-foot overlap has also been reported for cortical responses to movement observation and movement execution in parietal areas and also occipitotemporal areas [43], [44], [48]. Likely our experiment was not sensitive enough to detect differential responses to hands and feet. In contrast to previous studies, we did not use an independent functional localizer for each individual’s ‘hand-region’ but instead relied on anatomical coordinates from previous studies. This approach neglects common structural variance of brain function which in turn possibly affected our sensitivity to detect functional differences.

There are some additional points which need to be discussed. First, although we found a preference for hands and feet as compared to objects and faces in the left anterior intraparietal area in Experiment 1, we did not find a main effect for object category in Experiment 2 for this region of interest. However, we found a significant interaction between object category and orientation and significant preferential processing for orientations typically viewed for one’s own hand as compared to uncommon orientations as well as chairs. In Experiment 1 several orientations were shown in one block, whereas in Experiment 2 hand orientations were separated into different blocks. The fact that we did not find significant effects in anterior intraparietal areas comparing hands versus chairs across all orientations shown separately might be another indication that intraparietal areas specifically encode certain hand-orientations. Furthermore, other factors which might influence this pattern were that we used fewer categories, only one object exemplar and a smaller sample size in Experiment 2.

Secondly, previous studies suggest that areas encoding body-related visual information in the right occipitotemporal cortex are especially active when viewing bodies or body-parts from viewpoints typical for other people as compared to one’s own body [49], [50]. However, we did not find a significant interaction for category and orientation in the right occipitotemporal region of interest. Possibly, the orientations we used as uncommon for one’s own hand are also not very commonly viewed for other people as they involve a view of the hand from the top. Other differences include that previous studies looking at viewpoint specific processing employed stimulus sets including several body-parts and both body-halves, whereas our aim and stimulus design for Experiment 2 focused on investigated responses to one’s right hand only.

In conclusion, we found that regions in the left anterior intraparietal cortex and the left occipitotemporal cortex exhibited significant response preferences for viewing static images of hands and feet especially with orientations commonly viewed for one’s own body. Previously, it has been shown that these regions encode visual and multisensory events related especially to the space near the body. Furthermore, observing and executing hand movements also activates these brain areas. These areas might thus be dedicated to encoding sensory information which is used to process perceptual events closely related to one’s own body as well as to guide one’s own hand actions.

## Supporting Information

Table S1 Mean coordinates for ROI analysis obtained from previous studies.
(DOCX)
