# Supplementary material for: Preference for Orientations Commonly Viewed for One’s Own Hand in the Anterior Intraparietal Cortex
Source: PLoS One. 2013 Jan 7;8(1):e53812. doi: 10.1371/journal.pone.0053812 (PMC3538645; doi:10.1371/journal.pone.0053812)
Supplement: Table S1 — Mean coordinates for ROI analysis obtained from previous studies. (DOCX) [file pone.0053812.s001.docx]

| Publication | Left anterior intraparietal area | | | Right anterior intraparietal area | | | Left ventral premotor | | | Right ventral  premotor | | |
| --- | --- | --- | --- | --- | --- | --- | --- | --- | --- | --- | --- | --- |
| MNI coordinates (*) | x | y | z | x | y | z | x | y | z | x | y | z |
| Ehrsson et al. (2004)  Supplementary online material  Tables S1 and S2 | -33 | -56 | 66 | - | - | - | -58 | 15 | 11 | 48 | 17 | 43 |
| Makin et al. (2007)  Table 1, Average Experiment A-D | -40 | -40 | 44 | 39 | -42 | 45 | - | - | - | 53.3 | 9.7 | 16.3 |
| Brozzoli et al.  (2011)  Average whole brain and ROI-analyses (Table 1 and Table 2) | -26 | -54 | 56 | 30.7 | -44.7 | 54 | -50 | 0 | 40 | 40.7 | 7.3 | 28 |
| **Mean** | **-33** | **-50** | **55** | **35** | **-43** | **50** | **-54** | **8** | **26** | **47** | **11** | **29** |

Supplementary Table 1. Mean coordinates for ROI analysis obtained from previous studies.

*WFU Pickatlas (Maldjian et al. 2003) was used to convert from Talairach coordinates to MNI coordinates
